# Maternal risk factors for biliary atresia in neonates: a systematic review and meta-analysis

**DOI:** 10.3389/fped.2026.1817265

**Published:** 2026-04-07

**Authors:** Ke Zhang, Zhu Chen

**Affiliations:** Department of Pediatric Surgery, The Second People's Hospital, Yibin, Sichuan, China

**Keywords:** biliary atresia, meta-analysis, pregnancy or maternal factors, risk factors, systematic review

## Abstract

**Objective:**

The risk factors for neonatal biliary atresia remain unclear. This study aimed to systematically analyze maternal risk factors associated with the development of neonatal biliary atresia (BA).

**Methods:**

A systematic search was conducted in PubMed, Embase, Web of Science, and the Cochrane Library to retrieve relevant literature published from database inception to December 31, 2025, focusing on maternal risk factors for neonatal biliary atresia. Stata (Version 18.0) was used for meta-analysis.

**Results:**

A total of 10 retrospective studies were included in the meta-analysis. The results identified several statistically significant risk factors for the development of biliary atresia (BA), including maternal urogenital tract infection (OR = 1.21, 95% CI: 1.02–1.43), maternal diabetes mellitus (OR = 1.55, 95% CI: 1.20–2.00), preterm birth (OR = 2.64, 95% CI: 1.66–4.22), and low birth weight (OR = 1.66, 95% CI: 1.28–2.16). Additionally, the incidence of BA was significantly lower in neonates of White/Caucasian ethnicity (OR = 0.67, 95% CI: 0.46–0.98). By contrast, maternal smoking history, neonatal sex, maternal age (with 35 years as the cutoff), mode of delivery, and fetal number (singleton/multiple pregnancy) did not show statistically significant associations in this meta-analysis.

**Conclusion:**

The pathogenesis of neonatal biliary atresia is associated with multiple factors, including genetics, intrauterine infection, and maternal environmental exposures. Heightened vigilance for biliary atresia in neonates with high-risk characteristics is warranted to facilitate early diagnosis and treatment. However, due to the limited number of included studies, our findings require further validation through research with larger sample sizes.

## Background

Biliary atresia (BA) is a neonatal biliary tract disorder characterized by progressive inflammation and fibro-obliteration of the intrahepatic and extrahepatic bile ducts (1). Its etiology remains elusive to date, and the prevailing hypothesis postulates that viral infection occurring during the Pregnancy or maternal period or early postnatal stage triggers an autoimmune response targeting the biliary epithelial cells, ultimately leading to structural damage and obliteration of the bile ducts (2). BA is the leading cause of pediatric liver transplantation. Without timely surgical intervention and subsequent liver transplantation, affected infants will inevitably succumb to progressive cholestatic cirrhosis (3).

Although the Kasai portojejunostomy has ameliorated the natural disease progression of affected infants to a certain extent, its long-term prognosis remains suboptimal, with the majority of patients ultimately requiring liver transplantation. Consequently, from the perspectives of public health and clinical prevention, identifying the suspected risk factors for BA—particularly those potential factors occurring during pregnancy and the perinatal period that may be amenable to intervention—holds profound scientific value and practical significance. In recent years, numerous epidemiological studies have attempted to investigate the association between BA and various maternal factors; nevertheless, the findings of these studies have often been conflicting. For instance, while some studies have suggested that advanced maternal age or preterm birth may increase the risk of BA (4, 5), others have failed to confirm such associations (6, 7).

Against this backdrop, systematic reviews and meta-analyses, as the highest level of evidence for synthesizing existing data, providing more precise effect estimates, and identifying sources of heterogeneity, prove to be particularly necessary. Despite several meta-analyses examining selected risk factors associated with BA (8, 9), their limited analytical scope and insufficient recency preclude a comprehensive, systematic evaluation of evidence published in recent years. Specifically, a critical unaddressed gap remains in the holistic synthesis of multifactorial maternal risk factors. Therefore, this study aims to conduct a comprehensive meta-analysis to systematically evaluate the association between maternal factors and the risk of neonatal BA. By doing so, we seek to identify risk factors for BA and establish an evidence-based foundation for future etiological investigations and the identification of high-risk populations.

## Methods

### Literature search and inclusion criteria

In this systematic review, we adhered to the Meta-analysis of Observational Studies in Epidemiology (MOOSE) guidelines and the Preferred Reporting Items for Systematic Reviews and Meta-Analyses (PRISMA) statement (10). We performed a comprehensive systematic search in PubMed, Embase, Web of Science, and the Cochrane Library databases to retrieve relevant literature published from inception to December 31, 2025. The search strategy included terms such as “biliary atresia**”, “**congenital biliary obstruction”, “prenatal”, “maternal**”,** and **“**risk factor**”**, along with their corresponding free-text terms for expanded coverage. There were no limitations imposed on the type of eligible studies, but we only includes published literature in English, and does not include unpublished research or grey literature. Inclusion criteria were as follows: (i) studies that analyzed the impact of various maternal factors on the occurrence of neonatal biliary atresia; (ii) studies that provided at least one set of extractable, usable data. Duplicate publications, conference abstracts, review articles, reports without accessible full texts, and those lacking available data were excluded.

This research protocol has been registered on the PROSPERO platform, with the registration number being CRD420261321342.

### Data extraction and methodological quality assessment

At the first screening stage, Ke Zhang and Zhu Chen carried out a preliminary evaluation of the titles and abstracts for all retrieved studies; subsequently, both reviewers independently evaluated the full texts of potentially eligible studies. Any discrepancy arising during the process was resolved by consulting a third reviewer (Hongwen Ouyang), followed by group discussion until a consensus was reached. Both reviewers (Ke Zhang and Zhu Chen) independently extracted all relevant data, with subsequent comparisons performed to verify consistency. The following information was extracted from each study: first author's name, publication year, country, publishing journal, sample size, and the corresponding study period. The Newcastle–Ottawa Scale (NOS) was used to assess methodological quality, with a total score of 9 points. Studies with a score of less than 5 points were regarded as low-quality and thus excluded from the analysis (11).

After independent data extraction and subsequent integration by the two reviewers, 10 maternal factors were included in the meta-analysis, as follows: Outcome indicator: Neonatal biliary atresia (BA), diagnosed in accordance with the clinical and pathological criteria reported in each included study. Exposure factors and their unified definition criteria for extraction and analysis: 1. Neonatal sex: male/female. 2. Birth weight: low birth weight defined as <2,500 g; normal birth weight defined as ≥2,500 g. 3. Maternal age: advanced maternal age defined as ≥35 years; young maternal age defined as <35 years. 4. Mode of delivery: vaginal delivery/cesarean delivery. 5. Maternal diabetes mellitus: presence/absence of maternal diabetes (including gestational diabetes mellitus and pregestational diabetes). 6. Ethnicity: White/Caucasian/non-White (including Asian, African, Hispanic, etc.). 7. Maternal urogenital tract infection: presence/absence of maternal urogenital tract infection during pregnancy. 8. Preterm birth: presence/absence of preterm birth (defined as birth before 37 completed weeks of gestation in accordance with the standard definition in included studies). 9. Fetal number: singleton pregnancy/multiple pregnancy. 10. Maternal smoking history: presence/absence of maternal smoking during pregnancy (including active smoking in the first trimester or throughout pregnancy).

### Data synthesis and analysis

All statistical analyses were conducted in Stata software (Version 18.0). Heterogeneity was tested using the *I^2^* statistic and Q-test (*P* < 0.1 or *I^2^* > 50% was considered to imply statistical heterogeneity). A random-effects model was used for heterogeneity studies, and sensitivity analysis was performed to identify the source of heterogeneity. Conversely, a fixed-effects model was used to synthesize the data.

## Results

### Literature screening process and basic characteristics of included studies

**Figure 1:**
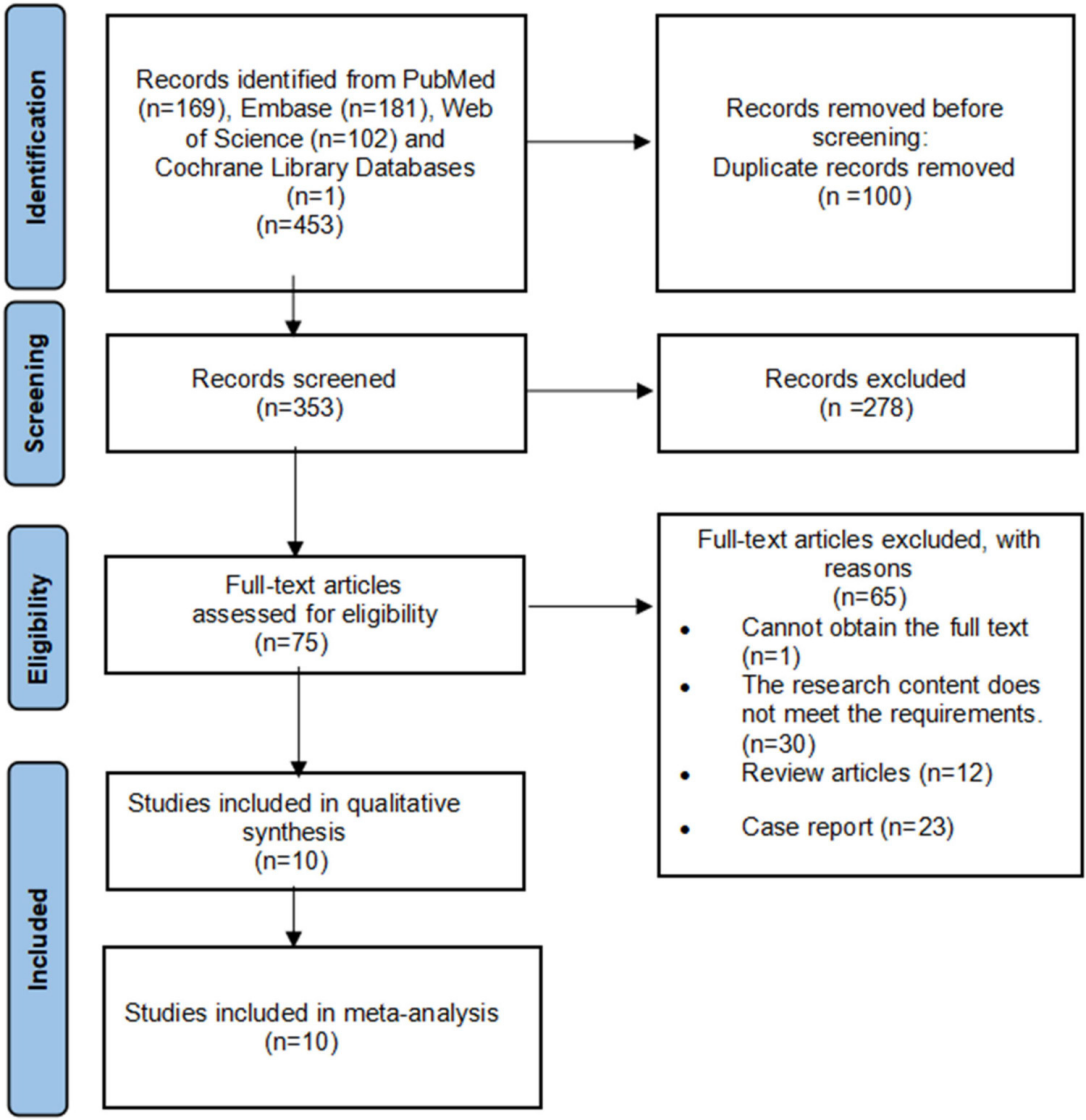
Flowchart of search strategy and study selection.

A total of 453 studies were initially retrieved, and 10 retrospective studies (4–7, 12–17) were ultimately included after screening. The combined sample size across all included studies was 7,564,811 pediatric participants, with 2306 in the BA cohort and 7,562,505 in the non-BA cohort. The screening process is shown in Figure 1. All 10 included studies had NOS quality scores ranging from 6 to 8 points (Table 1), confirming their methodological eligibility for this study. All the included studies were deemed eligible for the meta-analysis. Table 2 summarizes all the research results.

**Table 2:** Summary of research results.

Risk Factors	Number of research（n）	I^2^	OR	95%CI
Neonatal Sex	10	75.3%	0.86	0.70–1.04
Birth Weight	4	39.7%	1.66	1.28–2.16
Maternal Age	7	64.3%	0.77	0.59–1.02
Mode of Delivery	3	0%	1.14	1.00–1.30
Maternal Diabetes Mellitus	6	40.6%	1.55	1.20–2.00
Ethnicity	4	70.4%	0.67	0.46–0.98
Maternal Urogenital Tract Infection	3	0%	1.21	1.02–1.43
Preterm Birth	4	62.1%	2.64	1.66–4.22
Fetal Number	4	0%	0.70	0.46–1.07
Maternal Smoking History	3	12.6%	1.02	0.71–1.48

**Table 1:** Characteristics of studies included in meta-analysis.

Author	Year	Country	Journal	N	BA	Research time	NOS
Lin	2025	China	Digestive diseases and sciences	2783	253	2004–2016	7
Rita	2024	USA	Harmful algae	190	38	2001–2019	7
Wang	2024	China	JAMA network open	3359	447	2004–2020	8
Chang	2022	China	Pediatric research	1646859	285	2004–2017	6
Cavallo	2022	USA	The Journal of pediatrics	4689920	305	1999–2014	7
Jiang	2021	China	BMJ open	1275	594	2015–2016	6
Sumarno	2020	Indonesia	Indian Journal of Forensic Medicine and Toxicology	219	85	2010–2017	7
Carmichael	2018	USA	Birth defects research	11264	152	1997–2011	6
Natalie	2007	USA	American Journal of Medical Genetics Part A	4151	62	1997–2002	7
Fischler	2002	Sweden	Journal of Pediatrics	1204791	85	1987–1997	7

BA, biliary atresia.

### Neonatal sex and biliary atresia

**Figure 2:**
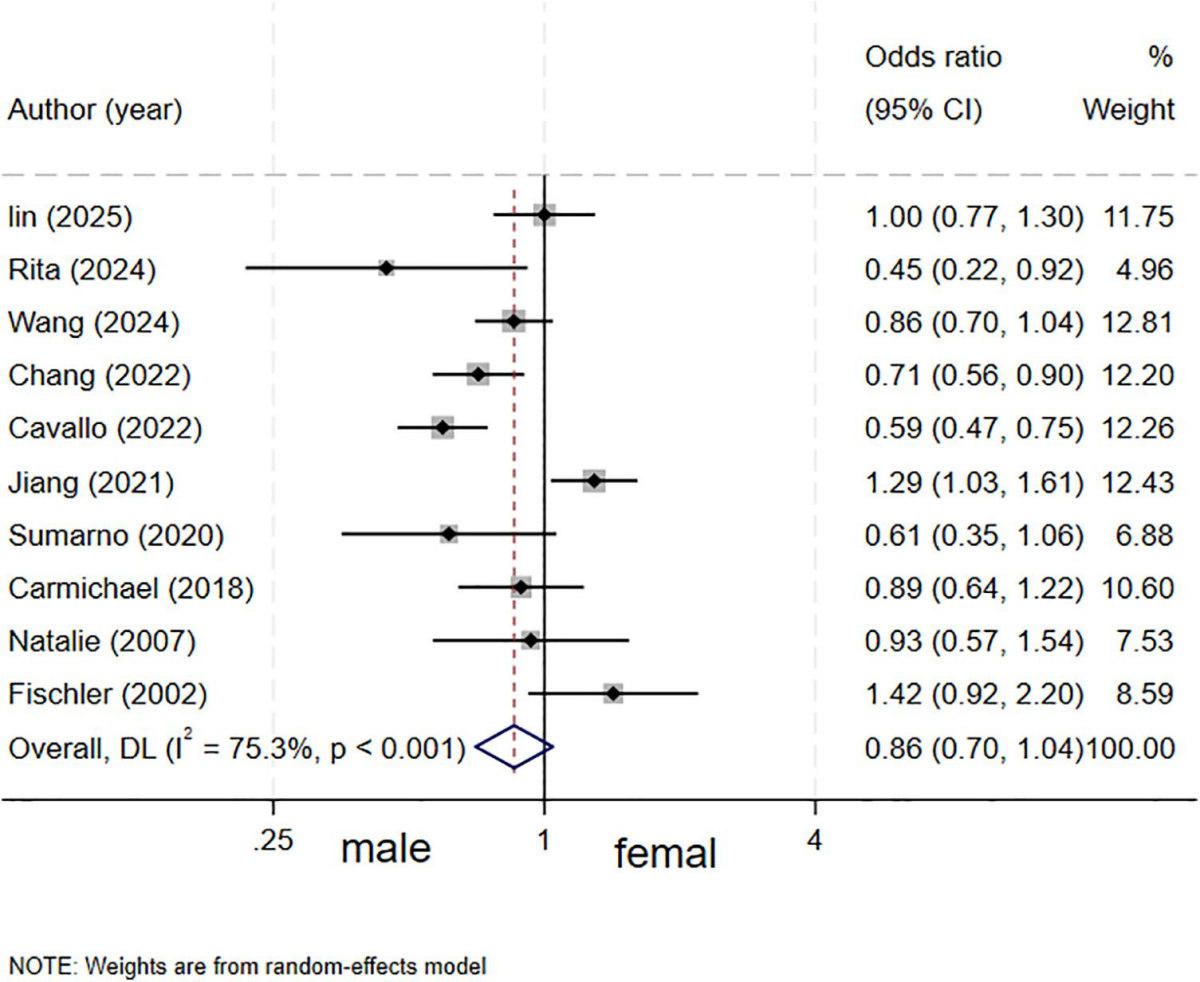
forest plot of neonatal sex and biliary atresia.

All ten studies (4–7, 12–17) assessed the association between neonatal sex and biliary atresia, and the results indicated that neonatal sex was not a risk factor for biliary atresia (OR = 0.86, 95% CI: 0.70–1.04; *I^2^* = 75.3%, *P* < 0.001) (Figure 2).

### Birth weight and biliary atresia

**Figure 3:**
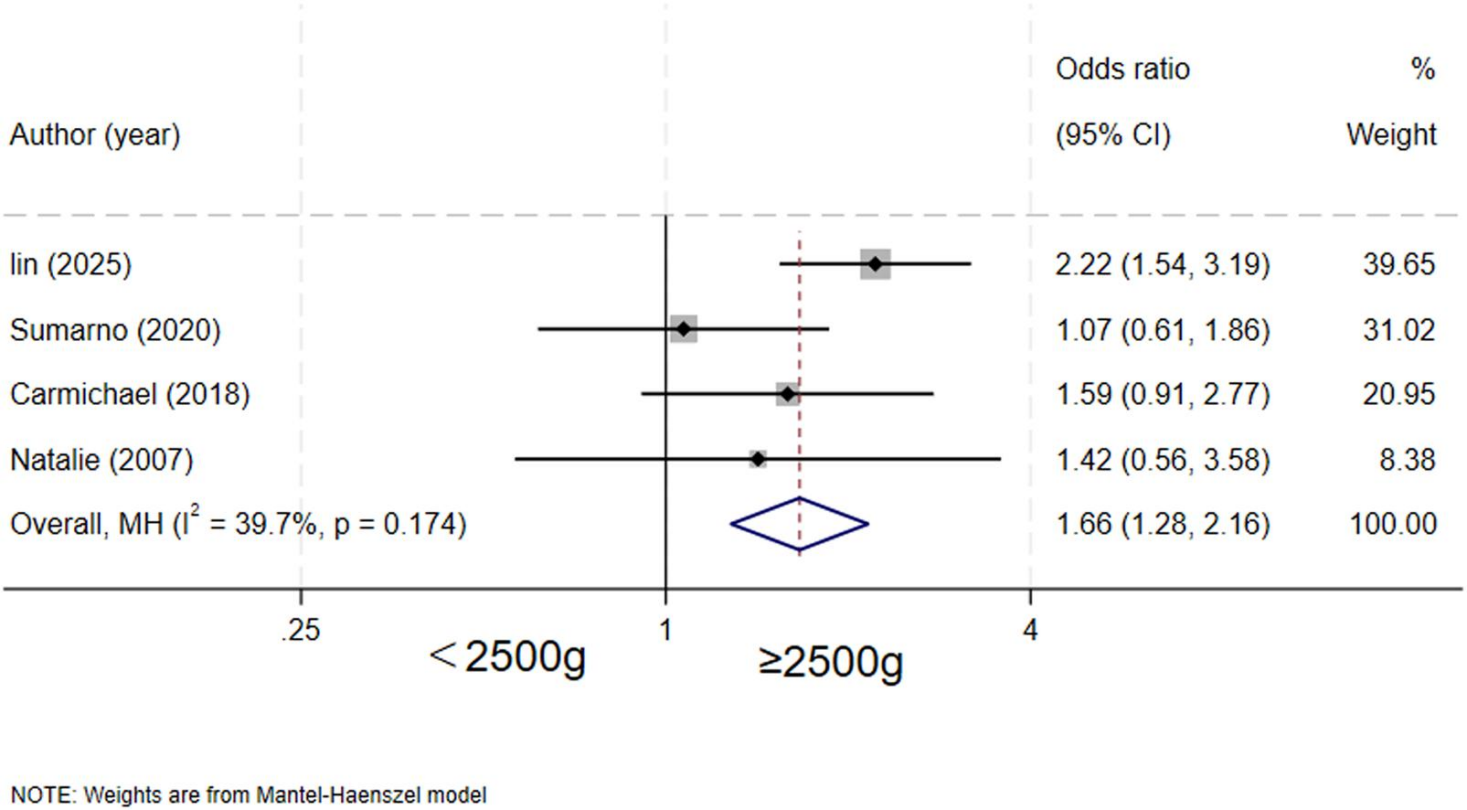
forest plot of birth weight and biliary atresia.

Four studies (5–7, 16) evaluated the association between birth weight and biliary atresia. The results demonstrated that low birth weight was associated with a statistically significant increased risk of biliary atresia (OR = 1.66, 95% CI: 1.28–2.16; I^2^ = 39.7%, *P* = 0.174) (Figure 3).

### Maternal age and biliary atresia

**Figure 4:**
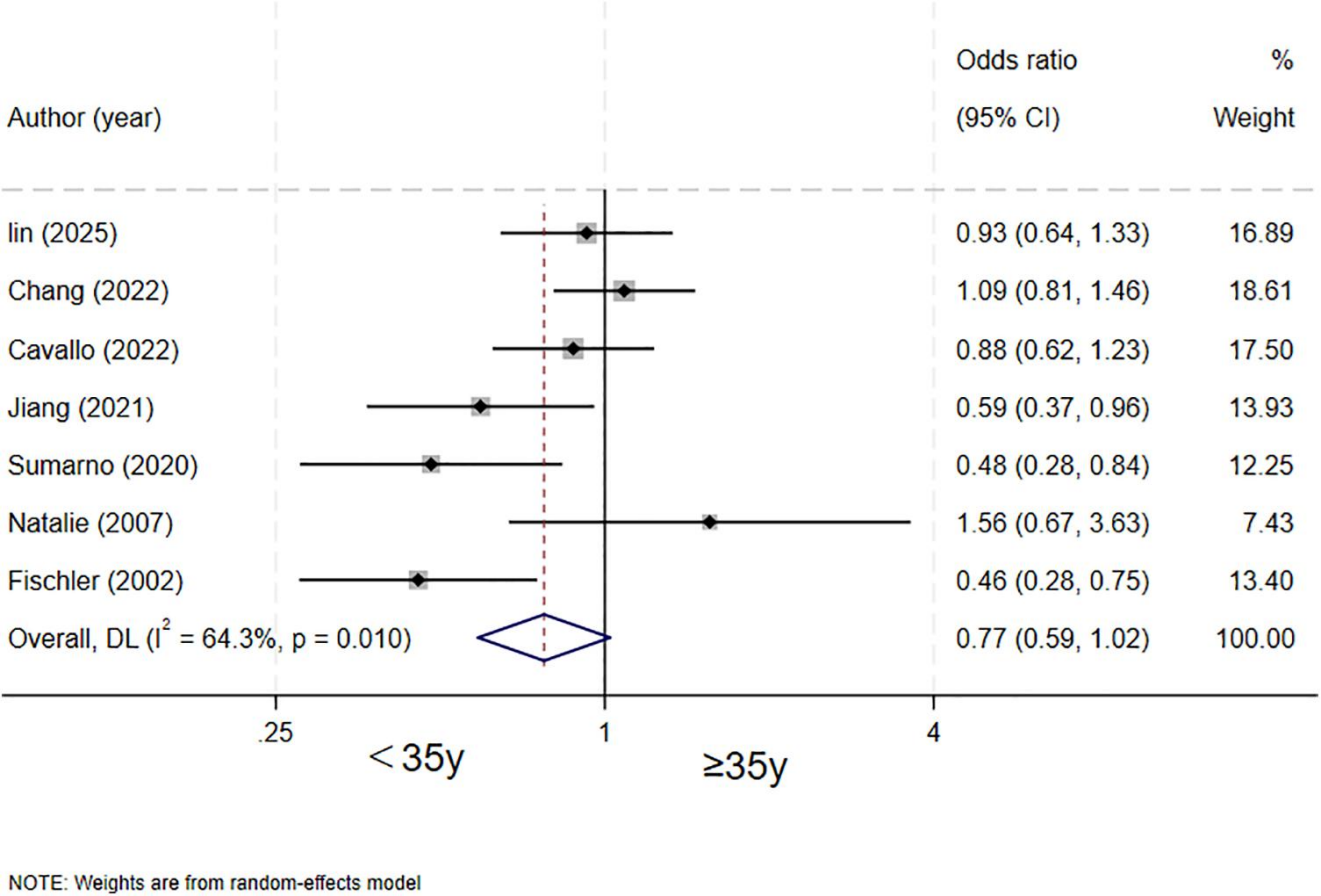
forest plot of maternal age and biliary atresia.

Seven studies (4–7, 14, 15, 17) evaluated the association between maternal age and biliary atresia. The results showed that maternal age was not a significant risk factor for biliary atresia (OR = 0.77, 95% CI: 0.59–1.02; *I^2^* = 64.3%, *P* = 0.01) (Figure 4).

### Mode of delivery and biliary atresia

**Figure 5:**
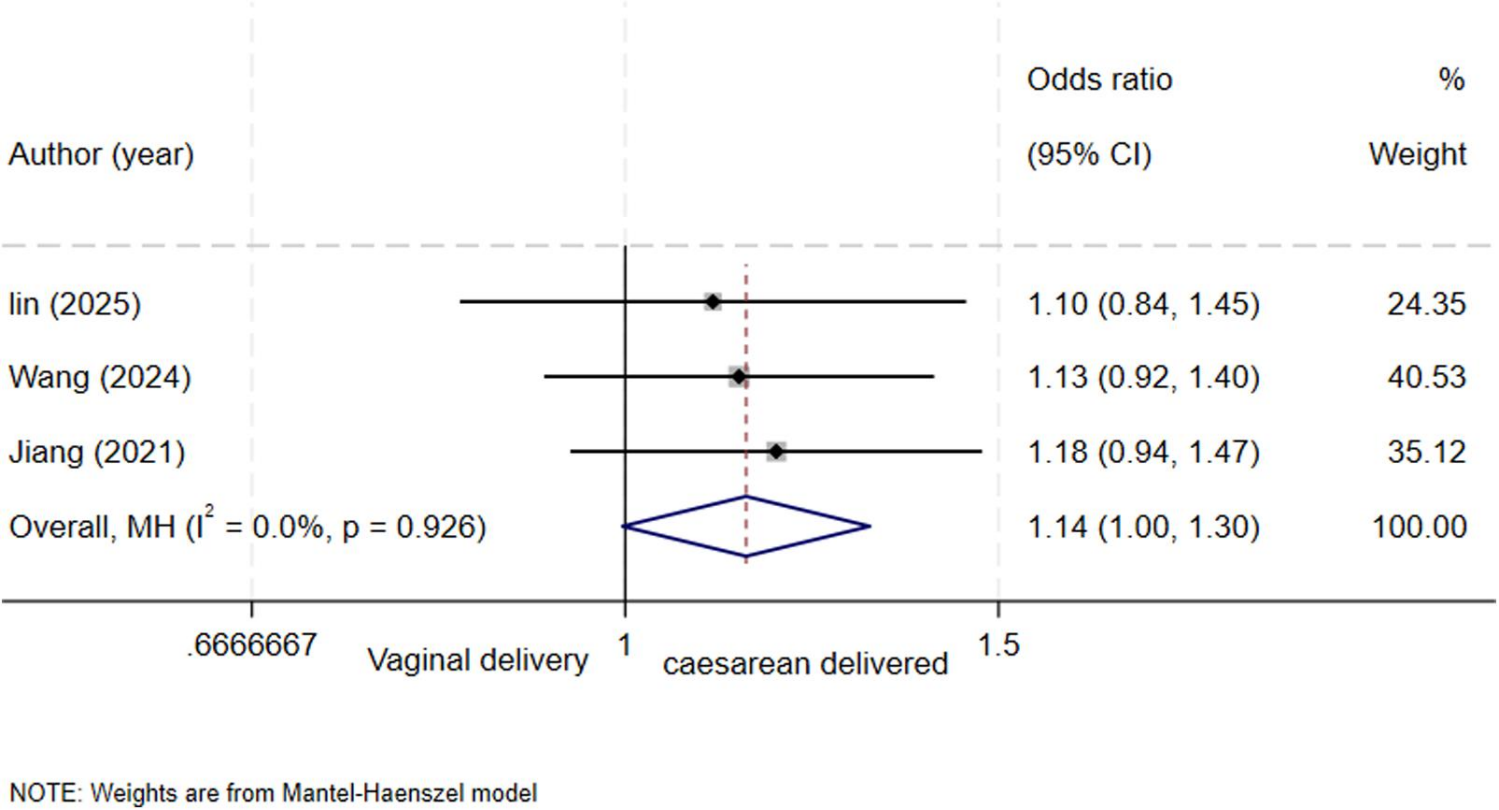
forest plot of mode of delivery and biliary atresia.

A total of three studies (4, 6, 13) assessed the association between mode of delivery and biliary atresia. The results showed that the mode of delivery was a borderline significant risk factor for biliary atresia (OR = 1.14, 95% CI: 1.00–1.30; *I^2^* = 0%, *P* = 0.926) (Figure 5).

### Maternal diabetes mellitus and biliary atresia

**Figure 6:**
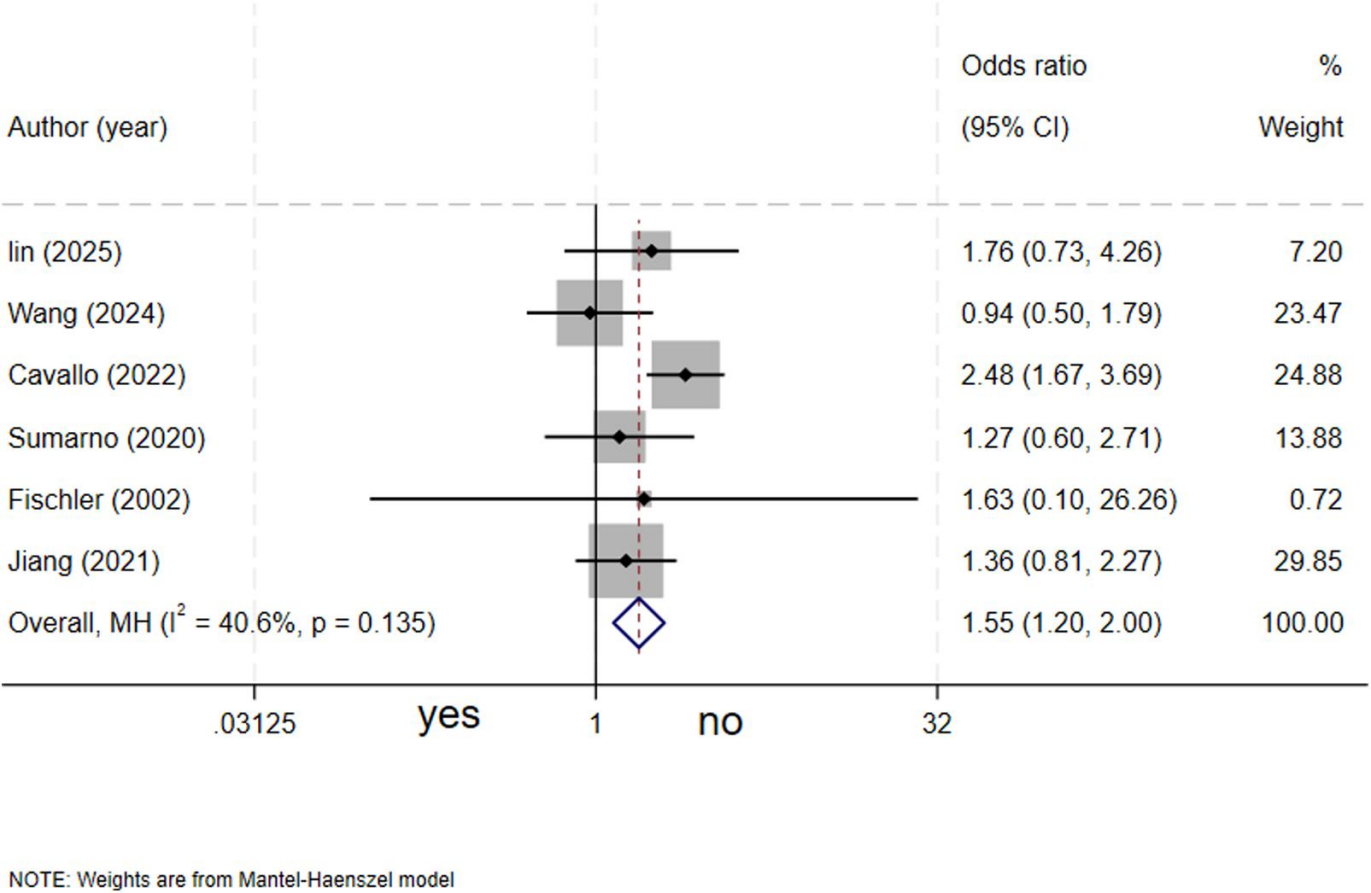
forest plot of maternal diabetes and biliary atresia.

A total of six studies (4–6, 13, 15, 17) assessed the association between maternal diabetes and biliary atresia. The meta-analysis revealed that maternal diabetes mellitus was associated with a significantly increased risk of biliary atresia in neonates (OR = 1.55, 95% CI: 1.20–2.00; *I^2^* = 40.6%, *P* = 0.135) (Figure 6).

### Ethnicity and biliary atresia

**Figure 7:**
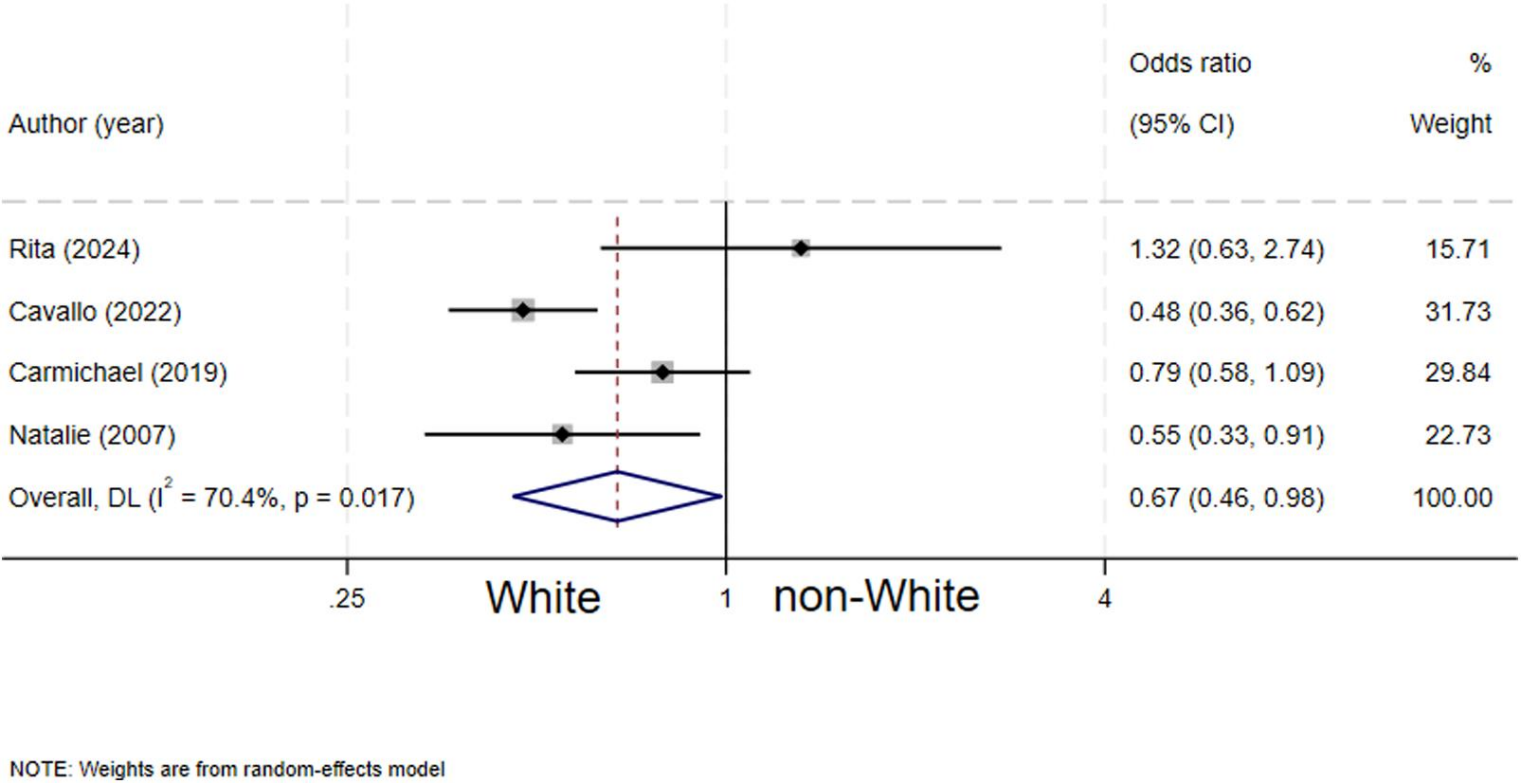
forest plot of ethnicity and biliary atresia.

Four studies (7, 12, 15, 16) evaluated the association between ethnicity and biliary atresia. The results showed that White/Caucasian ethnicity was associated with a lower risk of biliary atresia in neonates (OR = 0.67, 95% CI: 0.46–0.98; *I^2^* = 70.4%, *P* = 0.017) (Figure 7).

### Maternal urogenital tract infection and biliary atresia

**Figure 8:**
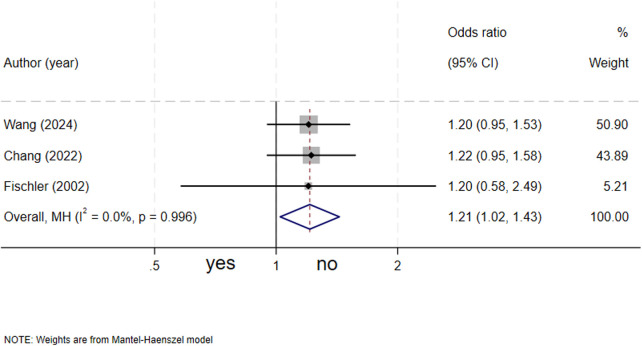
forest plot of maternal genitourinary tract infection and biliary atresia.

Three studies (13, 14, 17) evaluated the association between maternal genitourinary tract infection and biliary atresia. The meta-analysis demonstrated that maternal urogenital tract infection was a significant risk factor for biliary atresia in neonates (OR = 1.21, 95% CI: 1.02–1.43; *I^2^* = 0%, *P* = 0.996) (Figure 8).

### Preterm birth and biliary atresia

**Figure 9:**
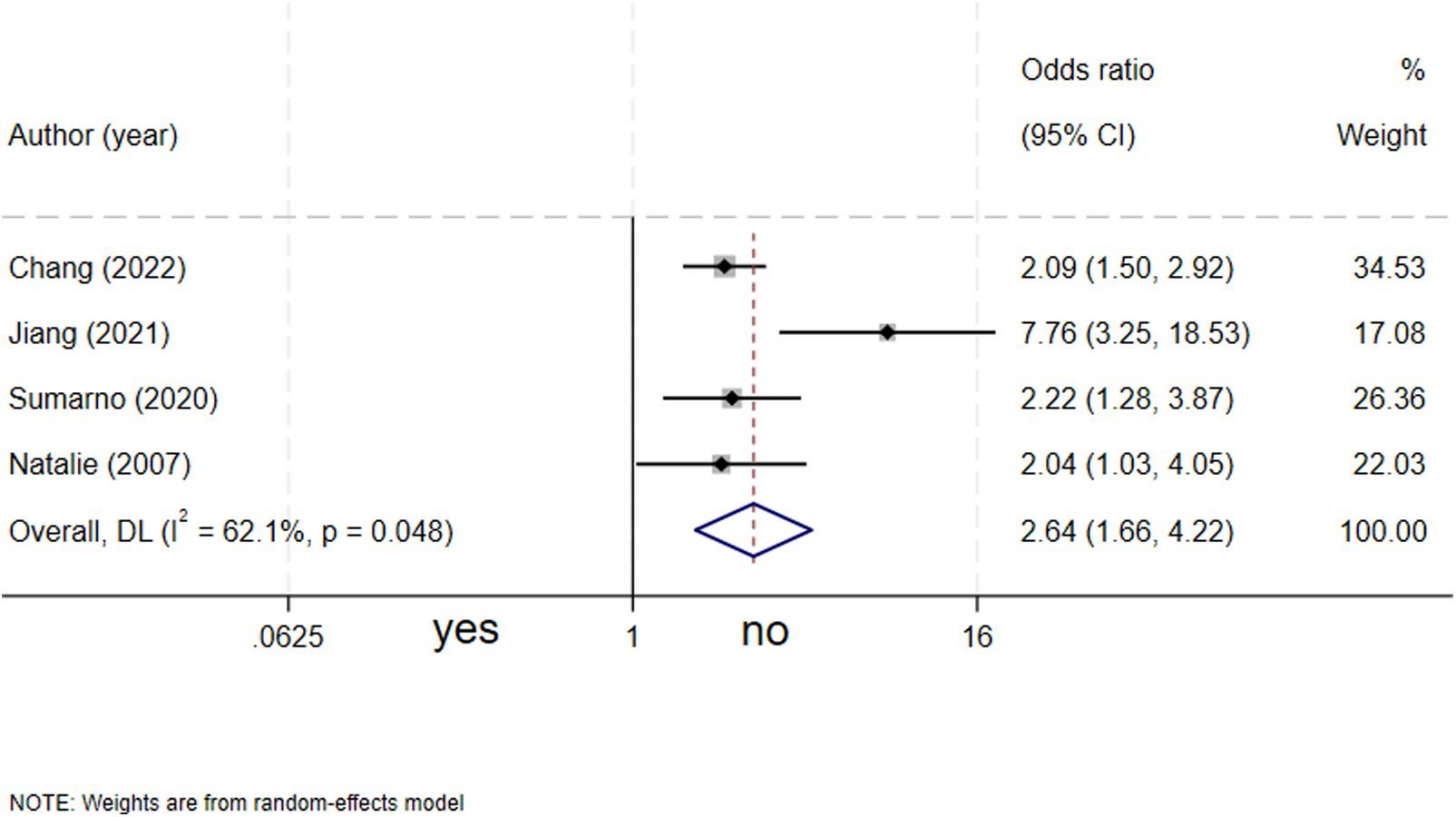
forest plot of preterm birth and biliary atresia.

A total of four studies (4, 5, 7, 14) assessed the association between preterm birth and biliary atresia. The results showed that preterm birth was a significant risk factor for biliary atresia in neonates (OR = 2.64, 95% CI: 1.66–4.22; *I*^2^ = 62.1%, *P* = 0.048) (Figure 9).

### Fetal number and biliary atresia

**Figure 10:**
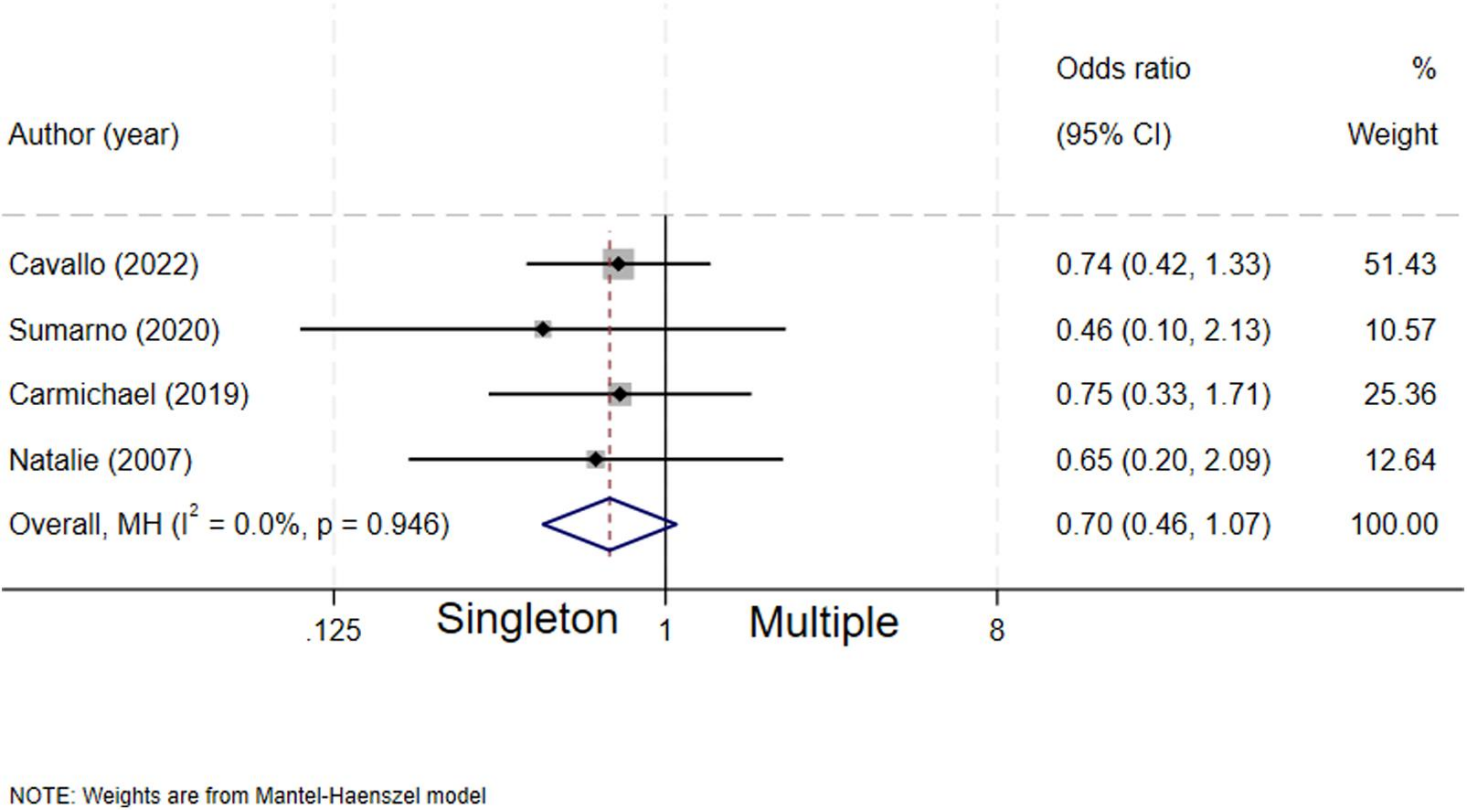
forest plot of fetal number and ailiary atresia.

A total of four studies (5, 7, 15, 16) assessed the association between the number of fetuses and biliary atresia. The results did not show a statistically significant association between fetal number (singleton/multiple pregnancy) and the risk of biliary atresia (OR = 0.70, 95% CI: 0.46–1.07; *I*^2^ = 0%, *P* = 0.946) (Figure 10).

### Maternal smoking history and biliary atresia

**Figure 11:**
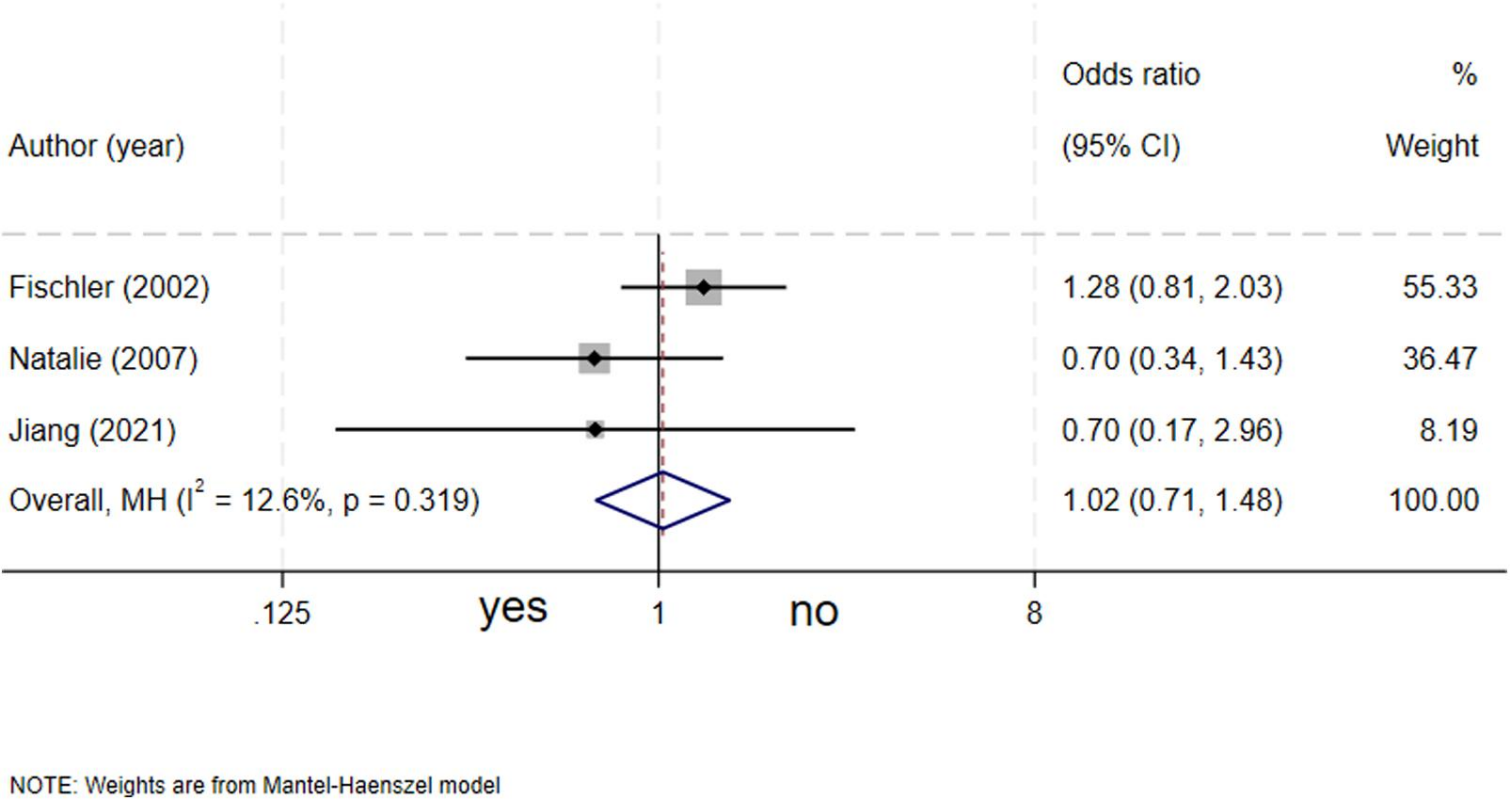
forest plot of maternal smoking history and biliary atresia.

Three studies (4, 7, 17) evaluated the association between maternal smoking history and biliary atresia. The results showed that maternal smoking history was not a significant risk factor for biliary atresia (OR = 1.02, 95% CI: 0.71–1.48; *I^2^* = 12.6%, *P* = 0.319) (Figure 11).

## Sensitivity analysis

**Figure 12:**
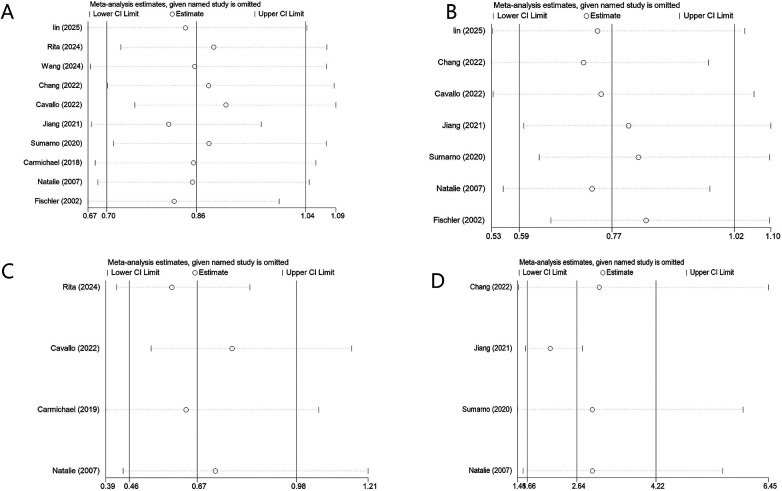
Sensitivity analysis for factors with significant heterogeneity. **(A)** sex, **(B)** gestational age, **(C)** race or ethnicity, **(D)** premature birth.

We conducted a sensitivity analysis on the factors with heterogeneity to assess the robustness of the results. The results indicated that no single study had a disproportionate influence on the overall effect size, suggesting that the findings were stable despite the presence of heterogeneity (Figure 12).

## Publication bias assessment

Egger's test was performed to assess the publication bias of all included factors. The results of Egger's test were as follows: the neonatal sex (*P* = 0.763), the birth weight (*P* = 0.338), the maternal age (*P* = 0.401), the mode of delivery (*P* = 0.588), the maternal diabetes (*P* = 0.452), the ethnicity (*P* = 0.39), the maternal urogenital tract infection (*P* = 0.882), the preterm birth (*P* = 0.317), the number of fetuses (*P* = 0.119), and the maternal smoking history (*P* = 0.466). All *P*-values were greater than 0.05, indicating that no significant publication bias existed in the present meta-analysis.

## Discussion

By synthesizing data from 10 studies (with the research period spanning from 1987 to 2016), this meta-analysis systematically evaluated the associations between a series of maternal factors and the risk of biliary atresia (BA) in neonates. Our principal findings revealed that maternal urogenital tract infection, non-Caucasian ethnicity, maternal diabetes mellitus, preterm birth, and low birth weight (<2,500 g) were identified as significant risk factors for the development of biliary atresia. In contrast, maternal smoking history, neonatal sex, maternal age (with 35 years as the cutoff), mode of delivery, and fetal number (singleton/multiple pregnancy) did not show statistically significant associations in this meta-analysis.

One of the principal conclusions of this study was a significant positive association between maternal urogenital tract infection (UTI) and an increased risk of BA. This finding carries important etiological implications. The maternal intestinal and urogenital microbiota play a pivotal role in the development of the offspring's immune system (18). Maternal infection may cause intrauterine infection through the hematogenous or ascending spread of pathogens. This can result in direct pathogen invasion of the developing fetal hepatobiliary system. Alternatively, pathogens can trigger fetal hepatobiliary inflammation by transplacentally releasing cytokines and inflammatory mediators, thereby interfering with the normal development and maturation of the fetal biliary tract (13, 19). Previous studies have indicated an association between specific infections such as cytomegalovirus and BA (8), while epidemiological evidence for generalized urogenital tract infections (UTIs) remains relatively limited. Our findings underscore the importance of routine screening and control of maternal infections. Future investigations are needed to elucidate the association between specific pathogens, the exact gestational age at infection onset, infection severity, and the risk of BA development.

Our analysis demonstrated that neonates of non-White ethnicities (including Asian, African, Hispanic, etc.) have a significantly higher risk of developing BA compared to neonates of White/Caucasian ethnicity. This finding is consistent with the geographic and ethnic distribution patterns of the global BA incidence, as well as the conclusions of previous studies (20); for instance, the incidence of BA is notably higher in East Asian regions (such as Taiwan Region of China and Japan) and specific ethnic groups (2, 13), which strongly suggests a central role of genetic susceptibility in the pathogenesis of BA. Polymorphic variations in genes linked to immune regulation, biliary tract development, toxin metabolism or anti-infective responses among different populations may underpin their differential susceptibility to environmental triggers. The meta-analysis by Li et al. (21) provided further genetic evidence showing that the ADD3 rs2501577 polymorphism is associated with an increased risk of BA, particularly in Asian populations. However, ethnicity itself represents a complex sociodemographic construct encompassing multiple confounding factors such as genetics, environment, culture, and socioeconomic status. The significant heterogeneity observed in this analysis (I^2^ = 70.4%) may precisely reflect the varying definitions of the non-White population across different studies and the unmeasured differences in environmental exposures within these populations. This finding underscores the necessity for future large-scale, cross-ethnic genome-wide association studies (GWAS) and rigorous investigations of gene-environment interactions (G × E).

Gestational diabetes mellitus (GDM) has been confirmed as an independent risk factor for BA. Prior studies have indicated that infants born to mothers with diabetes have an increased risk of congenital malformations (20). The intrauterine hyperglycemic environment may impair fetal organ development through multiple mechanisms, including increased oxidative stress, advanced glycation end product (AGE) formation, and inflammatory pathway activation, ultimately leading to DNA damage, microRNA (miRNA) dysregulation, and the perturbation of various signaling pathways, thereby disrupting normal fetal organ development (20, 22, 23). For example, the study by Kong et al. (24) found that high expression of the EDIL3 gene was significantly associated with the activation of the PI3K-AKT signaling pathway. The PI3K-AKT pathway is a crucial pathway in insulin signaling and may be aberrantly regulated in the intrauterine hyperglycemic environment caused by GDM. This finding suggests that the association between GDM and BA may not only involve oxidative stress and inflammatory pathways but may also interfere with the normal development of the fetal biliary system by affecting the expression of related genes such as EDIL3 and the regulation of the PI3K-AKT pathway. These mechanism studies have provided a new perspective for understanding the molecular basis of the increased risk of BA associated with GDM.

This finding carries direct public health implications, as it points to a potential modifiable factor—the optimization of pre-pregnancy and antenatal blood glucose management—which may help reduce the risk of multiple fetal developmental abnormalities, including BA. However, prospective cohort studies are warranted to validate whether strict blood glucose control can alter the risk of BA development.

Preterm birth and low birth weight were both associated with an elevated risk of BA in our analysis. These two factors are closely interrelated and frequently co-occur, potentially representing a fetus that has experienced an adverse, stressful intrauterine growth environment, which itself could be induced by infection, placental insufficiency, or other maternal morbidities such as diabetes (14). Accordingly, the observed associations between preterm birth, low birth weight, and BA may be partially mediated by the effects of other risk factors (e.g., infection, diabetes mellitus).

Notably, maternal smoking history, neonatal sex, advanced maternal age, cesarean section, and multiple pregnancy were not associated with a significant risk of BA in this meta-analysis.

This null finding for maternal smoking particularly merits in-depth discussion. Robust epidemiological evidence has established that maternal smoking is associated with a variety of fetal congenital malformations (25), and tobacco contains a multitude of well-characterized hepatotoxic and teratogenic substances (26). Offspring with prenatal tobacco exposure have an elevated risk of acute liver injury and metabolic dysfunction-associated steatotic liver disease (MASLD) during childhood or adulthood (26); accordingly, maternal smoking is theoretically a plausible risk factor for BA. However, our findings failed to support this hypothesis. A critical explanation for this discrepancy is the substantial paucity of included studies. Among the 10 studies included in this analysis, only 3 explicitly reported detailed smoking exposure data and provided results eligible for pooled analysis. The extremely small sample size may increase the risk of Type II error, which significantly undermines the credibility of the findings. Furthermore, reporting bias may be present in the documentation of maternal smoking behavior, and heterogeneous definitions of smoking across studies—including first-trimester or throughout pregnancy, and smoking intensity—have further heightened the measurement uncertainty. Therefore, the current null finding is insufficient to support the notion that maternal smoking is not a risk factor for BA. Given that smoking is a modifiable behavioral factor, elucidating its association with BA holds significant public health value. Additional high-quality studies are thus urgently needed to re-evaluate this exposure factor, with the employment of objective measurement indicators and meticulous documentation of smoking timing, frequency, and dosage.

With respect to additional null findings: The neonatal sex (male-to-female ratio) is frequently discussed in BA's epidemiological studies, with a preponderance of evidence indicating a higher incidence in female infants (1, 2). However, our pooled results did not reveal a significant sex difference. This may suggest that sex-related disparities in BA may vary across different populations and the subtypes of BA. The null finding for advanced maternal age (≥35 years) may reflect the effective management of pregnancy-related complications in older women within modern maternal care, which could attenuate its independent risk for BA.

The absence of significant associations for mode of delivery and fetal number (singleton/multiple pregnancy) with BA risk suggests that the etiology of BA may be rooted primarily in fetal developmental processes or intrinsic susceptibility, rather than in the intrapartum process itself or multiple gestation. This finding, to some extent, suggests that research focus should be further directed toward the fetal period rather than intrapartum events. However, the association between mode of delivery and BA was borderline significant, with a confidence interval that just touched the null value (1.00). While not definitive, the point estimate of 1.14 suggests a potential modest increase in risk among neonates delivered by cesarean section. This could reflect underlying confounding factors (e.g., maternal indications for cesarean section) rather than a direct causal effect. The borderline finding may also be due to limited statistical power, as only three studies were included in this analysis. Given the imprecision of this estimate, larger studies with better control for confounders are needed to clarify the relationship.

The heterogeneity testing in this study revealed significant heterogeneity across four factors: ethnicity, sex, preterm birth, and maternal age. Such clinical and methodological heterogeneity constitutes a common challenge in meta-analyses of observational studies, warranting cautious interpretation of the findings. A random-effects model was employed to estimate the pooled effect size in a more conservative manner. Sensitivity analyses failed to identify a specific source of the observed heterogeneity conclusively. Based on the literature and clinical context, we propose that the heterogeneity may arise from the following sources:

1. For ethnicity (*I*^2^ = 70.4%), the main sources of heterogeneity may include: 1) inconsistent definitions of “non-Caucasian” across studies—some studies categorized Asian, African, and Hispanic populations collectively as non-Caucasian, despite substantial differences in genetic backgrounds and environmental exposures among these groups; 2) population-specific genetic susceptibility to BA, such as the more pronounced effect of ADD3 gene polymorphisms in Asian populations (21); and 3) inadequate measurement or control of regional variations in environmental exposures (e.g., infectious disease profiles, dietary habits, and perinatal healthcare quality).

2. For maternal age (*I^2^* = 64.3%), heterogeneity may stem from: 1) varying degrees of adjustment for the collinearity between advanced maternal age and pregnancy complications (e.g., gestational diabetes mellitus, hypertension) across studies; and 2) improvements in perinatal healthcare over time, which may have mitigated the risk of adverse pregnancy outcomes in older mothers—the wide publication period of included studies (1987–2016) may reflect differences in clinical practice across different eras.

3. For preterm birth (*I^2^* = 62.1%), potential explanations for heterogeneity include: 1) differences in the definition of preterm birth—while some studies used 37 weeks of gestation as the cutoff, others employed more refined stratification (e.g., extremely preterm, moderate preterm); 2) etiological heterogeneity of preterm birth—infection-related preterm birth may have different effects on fetal development compared to non-infection-related preterm birth; and 3) differential interactions between preterm birth and other risk factors (e.g., infection, diabetes) across study populations.

There were several limitations in this meta-analysis: 1) With the exception of neonatal sex, most risk factors were analyzed based on a limited number of studies. This compromised the precision of the pooled effect sizes and the feasibility of subgroup analyses, and thus the stability of our conclusions awaits validation by additional studies. 2) All included studies were retrospective in design, and the potential impact of selection bias or information bias cannot be entirely ruled out. 3) As noted previously, the broad time span of included studies may lead to incomplete literature retrieval. 4) The definitions of both exposure factors and the outcome (BA subtypes) may have varied across included studies, which could have compromised the robustness of the findings. 5） Because this meta-analysis simultaneously evaluated 10 potential risk factors for biliary atresia, the risk of false-positive findings due to multiple comparisons should be considered. Therefore, our findings should be interpreted with caution, and confirmatory studies with pre-registered hypotheses and larger sample sizes are warranted.

The implications of the present study: Despite these limitations, the present meta-analysis still provides valuable directions for clinical practice and future research: 1) The findings suggest that for neonates with a history of maternal gestational diabetes mellitus (GDM) or urogenital tract infection (UTI), or those born preterm, with low birth weight, or of non-White ethnicity, pediatricians and neonatologists should heighten vigilance for cholestatic symptoms and consider including BA in the early differential diagnosis. This facilitates timely relevant examinations to achieve early diagnosis and treatment of BA in infants, thereby improving the prognosis. Additionally, pre-pregnancy counseling and antenatal care should be strengthened, with active prevention and treatment of GDM and UTI during pregnancy. 2) For factors with significant public health implications but insufficient evidence, such as maternal smoking, dedicated studies are warranted to conduct further verification. 3) For identified risk factors (e.g., UTI, GDM), animal models and cellular experiments should be used to clarify the specific molecular and immunological mechanisms by which they affect biliary tract development. 4) The findings of this study reinforce the imperative for early screening in high-risk neonates and provide a focused direction and evidence-based basis for research into the preventive strategies of BA.

## Conclusion

The pathogenesis of neonatal biliary atresia is associated with multiple factors, including genetics, intrauterine infection, and maternal environmental exposures. Heightened vigilance for biliary atresia in neonates with high-risk characteristics is warranted to facilitate early diagnosis and treatment. However, due to the limited number of included studies, our findings require further validation through research with larger sample sizes.
